# Bi-stable resistive switching characteristics in Ti-doped ZnO thin films

**DOI:** 10.1186/1556-276X-8-154

**Published:** 2013-04-04

**Authors:** Adnan Younis, Dewei Chu, Sean Li

**Affiliations:** 1 School of Materials Science and Engineering, University of New South Wales, Sydney, NSW, 2052, Australia

**Keywords:** Electrodeposition, Nanostructure, Resistive switching

## Abstract

Ti-doped ZnO (ZnO/Ti) thin films were grown on indium tin oxide substrates by a facile electrodeposition route. The morphology, crystal structure and resistive switching properties were examined, respectively. The morphology reveals that grains are composed of small crystals. The (002) preferential growth along *c*-axis of ZnO/Ti could be observed from structural analysis. The XPS study shows the presence of oxygen vacancies in the prepared films. Typical bipolar and reversible resistance switching effects were observed. High *R*_OFF_/*R*_ON_ ratios (approximately 14) and low operation voltages within 100 switching cycles are obtained. The filament theory and the interface effect are suggested to be responsible for the resistive switching phenomenon.

## Background

Resistance switching in metal oxide structures has attracted considerable attention because of its potential application to non-volatile memories [1-5]. Resistive random access memories (RRAMs) have many advantages over other technologies of data storage, such as much faster reading and writing rate, smaller bit cell size and lower operating voltages and very high retention time up to 10 years [2,6-8].

In general, the metal oxide thin films are prepared by physical methods, such as radio frequency magnetron sputtering and pulsed laser deposition, etc. It not only involves high fabrication cost but also limit the size and massive production. On the other hand, chemical methodologies, such as chemical bath deposition and hydrothermal, suffer from the problems of low crystallinity, disconnection of substrate and film or high-temperature calcinations. Compared with the aforementioned techniques, electrodeposition provides an effective way to fabricate high-quality metal oxide thin films at low temperature and ambient atmosphere. Moreover, in this process, the deposition of metal oxide layers on the substrate is driven by the external electric field. Therefore, it is facile to precisely control the layer microstructure by this method and further design heterostructures with novel functionalities.

To date, various methods including doping [9], interface engineering [10] and nanoparticle incorporation [11,12] were used to improve the performance of RRAM devices. The effects of Au and Pt nanoparticles embedded in ZrO_2_ and TiO_2_ oxide films have also been studied [12,13].

Among the resistance switching materials, ZnO is especially attractive for its several unique advantages, such as the coexistence of unipolar and bipolar switching behaviour [14,15], the larger high resistance state to low resistance state (HRS/LRS) window [16] and the transparent and flexible application aspects [6,17]. The doping method has already been adopted to optimize the switching performance of ZnO, including Mn, Co, Cu and Ga [15,16,18-20], but the switching properties were not as optimized as for practical applications. Very few studies of the electric conduction mechanism for Ti-doped ZnO films have been reported [21-23]. Since the ionic radius of titanium is smaller than that of the zinc, when titanium atoms doped into a ZnO lattice, they act as scattering centres/donors by providing two free electrons. However, only a small amount of doped Ti^4+^ could induce more electrons and avoid acting scattering centres [24]. Also, Ti-doped ZnO films have more than one charge valence state in comparison to that of the ZnO films doped with other Group III elements.

The Ti precursor in aqueous solution controls the hydrolysis process of Ti ions, and this reaction is very fast in conventional precursors, such as TiCl_4_. The coordination number of Ti is six; therefore, ammonium hexafluorotitanate is more stable, and thus, it is suitable to use as a dopant. In this present work, we find that ammonium hexafluorotitanate is the most suitable compound for Ti doping and for controlled structural morphology.

In this paper, a study has been carried out on resistance switching properties of Ti-doped ZnO, where the films were prepared by a simple electrochemical deposition method at low temperature. Ti dopants were introduced into ZnO in order to enlarge the memory window via increasing the resistivity of the high-resistance state.

## Methods

Electrodeposition was carried out using an Autolab 302 N electrochemical workstation (Metrohm, Utrecht, The Netherlands). A standard three-electrode setup in an undivided cell was used. ITO (indium tin oxide) (9.3 to 9.7 Ω, 1.1 mm × 26 mm × 30 mm, Asashi Glass Corporation, Japan) was used as the working electrode while platinum foil (0.2 mm × 10 mm × 20 mm) as the counter electrode. The distance between the two electrodes was 30 mm. The reference electrode was an Ag/AgCl electrode in a 4-M KCl solution, against which all the potentials reported herein were measured.

The ITO substrates were first cleaned by detergent, then rinsed well with ethanol and DI water and then electrodeposited in a solution of 0.1 M Zn (NO_3_)_2_·6H_2_O with 2% (NH_4_)_2_TiF_6_ at 1 mA for 30 min, at 75°C. The phase composition of the samples was characterized by X-ray powder diffraction (Philips X’pert Multipurpose X-ray Diffraction System with Cu Kα; Philips, Amsterdam, The Netherlands). The morphologies of the samples were observed by scanning electron microscopy (Nova Nano SEM 230, FEI, Hillsboro, OR, USA). To measure the electrical property of the films, Au top electrodes were patterned and deposited by sputtering using a metal shadow mask. Voltage–current curves of the films were measured using an Autolab 302 N electrochemical workstation controlled with Nova software (with a possible error in current and voltage values as ±5%; Nova Software, Chongqing, China). All measurements were repeated at least twice to confirm the results. During measurement, the working electrode and sensor electrode were connected to the top Au electrode, and the reference and counter electrode were connected to the ITO substrate.

X-ray photoelectron spectroscopy (XPS) was performed with an ESCALAB250Xi spectrometer (Thermo Fisher Scientific, Waltham, MA, USA) using a monochromatized Al K alpha X-ray source (hV) 1486.6 eV with 20 eV pass energy. Hall effect measurements were carried out by the Accent HL5500PC (Nanometrics, Milpitas, CA, USA). All measurements were performed at room temperature.

## Results and discussion

The electrochemical synthesis of ZnO is a four-step process: First, nitrate ions and H_2_O are electrochemically reduced at the surface of the working electrode, resulting in an increase in the local pH value in the vicinity of the electrode (Equations 1 and 2). Then, the increase in the local pH leads to the precipitation of zinc ions as zinc hydroxide (Zn(OH)_2_, Equation 3) at a suitable temperature, and Zn(OH)_2_ can be transformed into ZnO. In the presence of Ti^4+^, part of the Ti^4+^ ions can be incorporated into ZnO lattices.

(1) $$\begin{array}{cc} {\mathrm{NO}}_{3}^{-}+H_{2}O+2e^{-}\to {\mathrm{NO}}_{0}^{-}+2{\mathrm{OH}}^{-} & -0.20\;V\;\mathrm{vs}.\;\frac{\mathrm{Ag}}{\mathrm{AgCl}} \end{array}$$

(2) $$\begin{array}{cc} 2H_{2}O+2e^{-}\to H_{2}{\;+\;2\mathrm{OH}}^{-} & -1.05\;V\;\mathrm{vs}.\;\frac{\mathrm{Ag}}{\mathrm{AgCl}} \end{array}$$

(3) $${\mathrm{Zn}}^{2+}{\;+\;2\mathrm{OH}}^{-}\to \mathrm{Zn}{\left(\mathrm{OH}\right)}_{2}$$

(4) $$\mathrm{Zn}{\left(\mathrm{OH}\right)}_{2}\to {\mathrm{ZnO}+H}_{2}O$$

(5) $${\mathrm{TiO}}_{2}\overset{{\mathrm{ZnO}}_{x}}{\to }{\mathrm{Ti}}_{{\mathrm{Zn}}^{..}}+2e^{-}+{2O}_{O^{x}}$$

**Figure 1:**
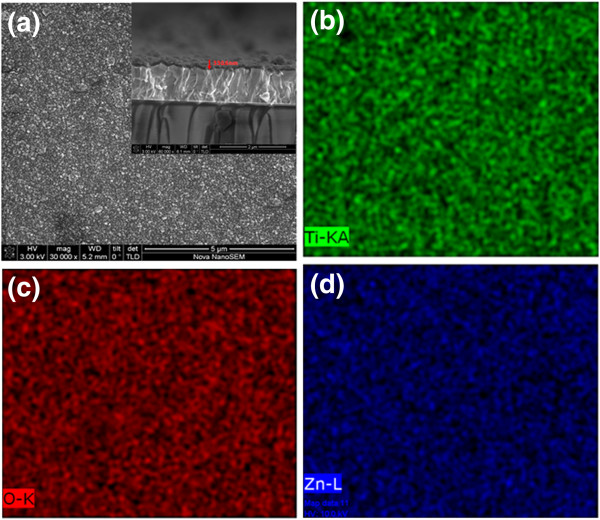
**The surface morphology of Ti-ZnO film.** (**a**) The SEM (inset cross-sectional image) and EDS mapping (**b**, **c** and **d**) images of Ti-ZnO films.

Figure 1a shows the SEM images of Ti-ZnO film. It is apparent that the grains are formed by many small crystallites aggregated with irregular shapes. In the inset of the same figure, a cross-sectional image was presented which shows film thickness as approximately 330 nm. EDS elemental maps are shown in Figure 1b,c,d. The O, Zn and Ti elemental maps have the same spatial distribution. This indicates a quite uniform distribution of elements in the synthesized products and demonstrates that the ZnO films are homogenously doped with Ti. The EDS spectra and element atomic percentage compositions were presented in the supporting information in Additional file 1: Figure S1.

**Figure 2:**
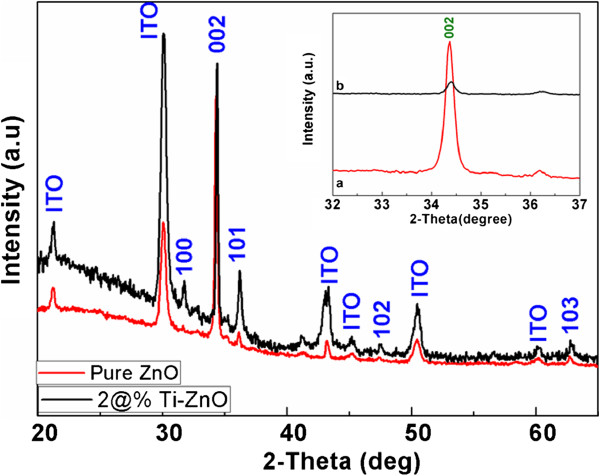
X-ray diffraction patterns of pure and 2% Ti-doped ZnO film (inset, magnified (002) peak).

The XRD pattern of the Ti-doped ZnO film (inset pure ZnO film) was displayed in Figure 2. The XRD patterns of the films are consistent with the hexagonal lattice structure, and a strong (002) preferential orientation is observed. It implies that the Ti atoms may substitute the zinc sites substitutionally or incorporate interstitially in the lattice. From Figure 2, it can be found that the locations of the diffraction peaks slightly shift towards higher diffraction angles, which illustrate the change in interplanar spacing (*d*-value). This is because of the different ionic radii between Ti^4+^ (0.0605 nm) and Zn^2+^ (0.74 nm); it is within the expectation that the diffraction peak position shifts, indicating that Ti^4+^ substitutes Zn^2+^ position in ZnO lattices.

**Figure 3:**
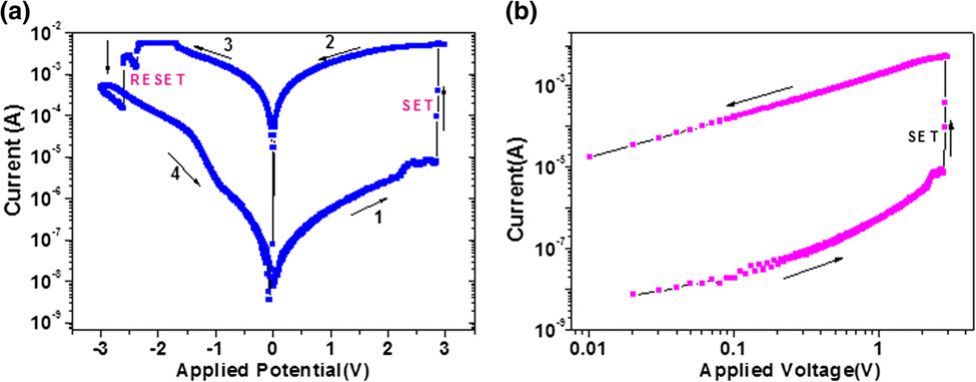
I-V curve of Au/ZnO/Ti/ITO is shown in the figure, (a) semi logarithmic scale and (b) log-log scale.

**Figure 4:**
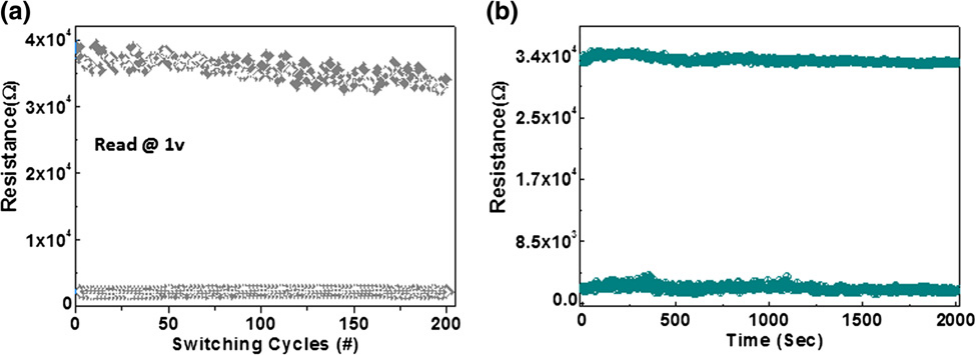
Memory performance, (a) endurance and (b) data retention performance of the 2% Ti@-ZnO.

The typical I-V characteristics of RRAM cell based on the Au/2% Ti-ZnO/ITO was carried out by sweeping voltage and at a speed of 0.01 V/s, in the sequence of 0→3→0→−3→0 V as shown in Figure 3a. During the measurements, the bias voltages were applied on the TE with BE grounded, and neither a forming process nor a current compliance was necessary for activating the memory effort. For the Ti-doped ZnO sample, with the increase of positive voltage, a significant change of resistance from the HRS to the LRS was observed at about 2.9 V, which is called the ‘set’ process. Subsequently, an opposite ‘reset’ process could also be seen when sweeping the voltage reversely to negative values, as evidenced by a two-step switching from LRS to HRS (Figure 4a). The first switching occurs at approximately −2.3 V (with I_RESET_ as 5.7 mA), and the second switching takes place at approximately −2.7 V (with I_RESET_ as 0.17 mA), after the resistance of the cell stays in an intermediate state for a short while. The multistage reset process observed in our sample might be due to the ruptures of multifilaments with different threshold potentials (*V*_th_). This phenomenon also gives rise to the concept of multilevel data storage as long as an effective control for *V*_th_ could be realized. The resistive switching behaviour of our sample exhibits a typical bipolar nature, that is, the sample device can only be written with a positive bias and erased with a negative one, as this happened in our sample device during numerous measurements.

For more understanding of the conduction and switching mechanisms of the memory device, the I-V characteristics are replotted in a log-log scale. Figure 3b shows the logarithmic plot of the previous I-V curve for the positive voltage sweep region, while it is similar for the negative branch. The I-V curve in LRS clearly shows an ohmic behaviour, which might be due to the formation of conductive filaments in the device during the set process. However, the conduction mechanism in off state is much more complicated. The charge transportation in this region is in agreement to the classical trap-controlled space-charge-limited conduction (SCLC), which consists of three regions: the ohmic region (*I* ∝ *V*), the Child's law region (*I* ∝ *V*^2^) and the steep current increase region [25]. The totally different conduction behaviours in these two states (LRS and HRS) also suggest that the high conductivity in on-state device should be a confined, filamentary effect rather than a homogenously distributed one. This indicates the fact that the active medium is much smaller than the device size, providing a potential of scaling.

The endurance characteristics of the Au/ZnO: Ti/ITO memory cell are shown in Figure 4a. The memory window defined by the two resistance states, i.e., (*R*_OFF_ − *R*_ON_) / *R*_ON_ ≈ *R*_OFF_/*R*_ON_, is more than 14. This is a high memory margin, making the device circuit very easy to distinguish the storage information between ‘1’ and ‘0’. The resistance of the HRS scatter in a certain extent during cycling. However, due to high *R*_OFF_/*R*_ON_ ratio of the present device, this kind of scattering may be tolerated. It can be seen that the memory margin keeps beyond 14 times during cycling, and the cell shows little degradation after 100 repeated sweep cycles. The endurance measurements ensured that the switching between on and off states is highly controllable, reversible and reproducible. After the device was switched on or off, no electrical power was needed to maintain the resistance within the given state.

To further demonstrate the stability of the resistive switching properties, data retention was gauged by examining the current level of the device in the on state over a long period of time (>2000 s) in air ambient. In this case, no appreciable change in resistance ratio (HRS/LRS) was observed in these devices, as shown in Figure 4b, while the information storage in these devices is likely to persist for an even longer time judging from the present trend of data.

The current–voltage measurements of pure ZnO sample were also performed and presented in the supporting information in Additional file 1: Figure S2. The memory margin of the device with 2% Ti@-ZnO was much better than the standard device (pure ZnO) as shown in Additional file 1: Figure S3. We also did perform the same measurements for both devices (pure and 2% Ti@-ZnO) without gold top electrode to see the possible effect of top electrode (results not shown here). Interestingly, both devices exhibited almost the same results as with the gold top electrode suggesting that gold top electrode is not playing critical/dominating role in resistive switching characteristics of these devices.

**Figure 5:**
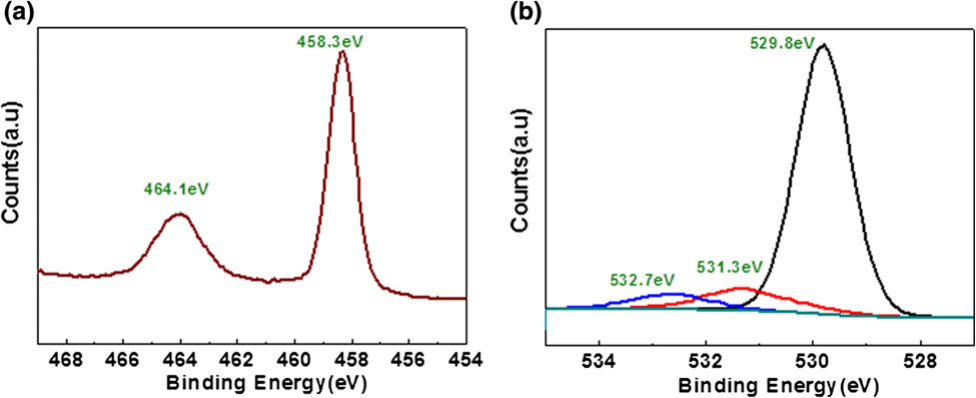
**XPS (a), Ti 2p and (b) O 1 s spectra of 2% Ti-doped ZnO film.**

The XPS measurements were carried out to investigate the surface chemical compositions and bonding states of the as-prepared sample. XPS analysis done on this sample shows the presence of Ti along with Zn and O. The binding energies of Ti 2p_3/2_ and 2p_1/2_ in ZnO/Ti are approximately 458.3 and approximately 464.1 eV, in agreement with the reported tetrahedral (Ti^4+^), as shown in Figure 5a [26]. Hence, tetravalent Ti may be replacing two divalent Zn atoms in ZnO forming a solid solution of 2% Ti-doped ZnO. Three peaks at 529.8, 531.3 and 532.7 eV can be observed in O 1 s XPS spectra (Figure 5b). The peak at 529.8 may be the character spectra of oxygen in ZnO structure [27]. The little oxygen peak at 531.3 eV can be assigned to the oxygen in TiO_2_[28], whilst the O peak at 532.7 may be attributed to chemisorbed oxygen ions present in the sample [26]. The chemisorbed oxygen impurities could be O^2−^, O^−^, O_2_^−^, O_2_^2−^ and OH^−^ ions as well [29,30], so the binding energy not only depends on the charge of oxygen species but also depends upon the crystallographic orientation of the bounded surface to which the oxygen atoms or molecules are bound [29], which points to the nonstoichiometric nature and presence of oxygen vacancies present in the film. Also, our synthesis method is a solution-based method, so these oxygen vacancies can easily be generated during growth process. From previous reports, it was believed that electrochemical migration of oxygen vacancies is the dominating factor in the resistive switching behaviour [31,32]. So we can also expect that the oxygen-deficient nature of the film which contains oxygen vacancies initially will enhance the resistance switching nature of prepared 2% Ti@-ZnO film.

In our recent study [33], the resistive switching characteristics of pure ZnO were improved (on/off, approximately 7) with Co doping in ZnO. In the present report, with the addition of Ti in ZnO, the resistive switching characteristics were further improved with on/off ratio (>14) and data retention time of 2,000 seconds was achieved.

## Conclusions

Ti-doped ZnO thin films were prepared by a facile electrochemical deposition method. The SEM, XPS and EDS mapping indicates that Ti is homogenously doped in ZnO films. The Ti-doped ZnO film had a similar structure to that of the pure ZnO film and had a preferential orientation in the (002) direction. The prepared film exhibits excellent resistance switching behaviour with a HRS/LRS ratio of about 14 during endurance test, much better than pure ZnO. In addition, the dominant conduction mechanism of LRS and HRS were explained by trap-controlled space-charge-limited conduction. The present work demonstrates that Ti doping can further enhance switching characteristics of pure ZnO films and thus have the potential for next-generation non-volatile memory applications.

## Competing interests

The authors declare that they have no competing interests.

## Authors’ contributions

AY and DC carried out the sample preparation, participated on its analysis, performed all the analyses, and wrote the paper. SL guided the study and participated in the paper correction. All authors read and approved the final manuscript.

## Supplementary Material

Additional file 1: Figures S1 to S3 Figure S1: EDS elemental spectrum of 2% Ti-doped ZnO (inset table represents atomic percentages). Figure S2: I-V curve of Au/ZnO/ITO (a) linear scale (b) semi logarithmic scale. Figure S3: Endurance performance of the pure ZnO. Click here for file
