# Supplementary material for: Bi-stable resistive switching characteristics in Ti-doped ZnO thin films
Source: Nanoscale Res Lett. 2013 Apr 4;8(1):154. doi: 10.1186/1556-276X-8-154 (PMC3623890; doi:10.1186/1556-276X-8-154)
Supplement: Additional file 1: Figures S1 to S3 — Figure S1: EDS elemental spectrum of 2% Ti-doped ZnO (inset table represents atomic percentages). Figure S2: I-V curve of Au/ZnO/ITO (a) linear scale (b) semi logarithmic scale. Figure S3: Endurance performance of the pure ZnO. [file 1556-276X-8-154-S1.docx]

# Bi-stable Resistive Switching Characteristics in Ti doped ZnO thin films

Adnan Younis, Dewei Chu^[[1]](#footnote-1)^* and Sean Li

*School of Materials Science and Engineering, University of New South Wales, Sydney, 2052, NSW, Australia*

**Supporting Information**

**Figure S1:** EDS elemental spectrum of 2% Ti-doped ZnO (inset table represents atomic percentages)

**Figure S2:** I-V curve of Au/ZnO/ITO (a) linear scale (b) semi logarithmic scale

**Figure S3:** Endurance performance of the pure ZnO.

1. *Corresponding Author, Tel.: +61 (0)2 9385 9934; Fax: +61 (0)2 9385 6565

   E-mail address: d.chu@unsw.edu.au [↑](#footnote-ref-1)
